# Molecular epidemiology and antimicrobial susceptibility of *Pseudomonas* spp. and *Acinetobacter* spp. from clinical samples at Jimma medical center, Ethiopia

**DOI:** 10.3389/fmicb.2022.951857

**Published:** 2022-09-20

**Authors:** Tsegaye Sewunet, Daniel Asrat, Yimtubezinash Woldeamanuel, Abraham Aseffa, Christian G. Giske

**Affiliations:** ^1^Division of Clinical Microbiology, Department of Laboratory Medicine, Karolinska Institutet, Stockholm, Sweden; ^2^Department of Microbiology, Immunology and Parasitology, Addis Ababa University, Addis Ababa, Ethiopia; ^3^Armauer Hansen Research Institute, Addis Ababa, Ethiopia; ^4^Department of Clinical Microbiology, Karolinska University Hospital, Stockholm, Sweden

**Keywords:** ESBLs, carbapenemase, *bla*_CTX–M–15_, *bla*_GES–11_, *bla*_NDM–1_, *P. aeruginosa*, *A. baumannii*, Ethiopia

## Abstract

**Introduction:**

Pseudomonas aeruginosa (*P. aeruginosa*) and *Acinetobacter baumannii* (*A. baumannii*) can cause difficult-to-treat infections. We characterized molecular epidemiology of ceftazidime-resistant *P. aeruginosa* and carbapenem-resistant *A. baumannii* at a tertiary hospital in Ethiopia.

**Materials and methods:**

Non-fermenting gram-negative bacilli (*n* = 80) isolated from admitted patients were subjected for species identification by MALDI-TOF. *Pseudomonas* species resistant to ceftazidime or meropenem, and *Acinetobacter* species resistant to meropenem, or imipenem were selected for whole genome sequencing. DNA extracted with EZ1 Advanced XL instrument (Qiagen, Hilden, Germany) was sequenced on Illumina (HiSeq2500) using libraries prepared by NEXTRA-kits (Illumina). Raw reads were assembled using SPAdes 3.13.0, and assembled genomes were used to query databases for resistome profile and sequence types.

**Result:**

Among *Pseudomonas* species isolated, 31.7% (13/41), and 7.3% (3/41) were non-susceptible to ceftazidime, and meropenem, respectively. Carbapenem-resistance was 56.4% (22/39) among *Acinetobacter* species. Moreover, 92% (12/13) of *Pseudomonas* species non-susceptible to ceftazidime and/or meropenem, and 89.4% (17/19) of *Acinetobacter* species encoded multiple resistance genes for at least three classes of antimicrobials. The prevalent β - lactamase genes were *bla*_OXA–486_ (53.8%, 7/13), *bla*_CTX–M–15_ (23.0%, 3/13) among *Pseudomonas*, and *bla*_GES–11_ (57.8%, 11/19) among *Acinetobacter*. The *bla*_OXA–51_-like β - lactamase, *bla*_OXA–69_ (63.1%, 12/19) was the most prevalent carbapenemase gene among *Acinetobacter* isolates. Single isolates from both *P. aeruginosa*, and *A. baumannii* were detected with the *bla*_NDM–1_. Sequence type (ST)1 *A. baumannii* and ST274 *P. aeruginosa* were the prevalent sequence types. A cgMLST analysis of the ST1 *A. baumannii* isolates showed that they were closely related and belonged to the international clonal complex one (ICC1). Similarly, ST274 *P. aeruginosa* isolates were clonally related.

**Conclusion:**

The prevalence of MDR isolates of *Pseudomonas* and *Acinetobacter* spp. was high. *A. baumannii* isolates were clonally spreading in the admission wards at the hospital. Emergence of *bla*_NDM–1_ in the intensive care, and surgical wards of the hospital is a severe threat that requires urgent intervention.

## Introduction

*Pseudomonas aeruginosa (P. aeruginosa)* and *Acinetobacter baumannii (A. baumannii)* are among the main causes of nosocomial infections (De Oliveira et al., 2020). They belong to the group of bacteria known as “ESKAPE” (*Enterococcus faecium, Staphylococcus aureus, Klebsiella pneumoniae, A. baumannii, P. aeruginosa*, and *Enterobacter* species). Moreover, these group of bacteria are difficult to treat in most cases (Bergogne-Bérézin and Towner, 1996; De Oliveira et al., 2020; Ma et al., 2020).

The prevalence of antimicrobial resistant sub-populations of these strains has rapidly increased over the last few decades (Wong et al., 2017; Horcajada et al., 2019). Carbapenemase-producing *A. baumannii* (CRAB) and *P. aeruginosa* were listed top two of the three critical priority pathogens for which new antimicrobials are urgently needed (WHO, 2017). Several *Acinetobacter* and *Pseudomonas* species were previously reported from different clinical samples from both animal, and human infections (Chusri et al., 2014; Wong et al., 2017; Agnese et al., 2018). Many of them were resistant to multiple classes of antibiotics primarily by several intrinsic resistance mechanism they encode, and secondly by acquired resistance mechanisms (Bello-López et al., 2020; Meng et al., 2020). Studies have also shown that multidrug-resistant strains of *P. aeruginosa* and *A. baumannii* were the main drivers of hospital-acquired infections (Djahmi et al., 2014; Eichenberger and Thaden, 2019; Kazmierczak et al., 2020). A recent review of global epidemiology of carbapenemase-producing Gram-negative bacteria reported that carbapenemase-producing *P. aeruginosa* (CRPA) were associated with high mortality and morbidity among hospitalized patients with pneumonia and bloodstream infections in the United States (Brink, 2019). Though regional variations are common, colonization by carbapenem-resistant *A. baumannii* increased the risk of acquisition of bloodstream infection four-fold (Munoz-Price et al., 2016; Bassetti et al., 2017). In low-income countries like Ethiopia, comprehensive microbiological data is lacking.

The classical phenotyping methods commonly used in low-income countries cannot reliably define mechanism of resistance in both *Pseudomonas* and *Acinetobacter* species. Lack of sufficient standardized genotypic methods for detection and tracking of multidrug-resistant or extensively drug-resistant isolates was one of the challenges to understand epidemiology of antimicrobial resistance in sub-Saharan African countries (Eichenberger and Thaden, 2019). In most cases, global reports on antimicrobial resistance lack data from African countries. Despite discrepancies in availability of data, there is sufficient overall evidence that carbapenemase-producing Gram-negative bacilli have become a threat to global health. Rapid detection and tracking of any ongoing spread of resistant strains is necessary.

We aimed to analyze the phenotypic and molecular characteristics of *Pseudomonas* species and *Acinetobacter* species isolated from clinical samples at Jimma Medical Center (JMC), a tertiary hospital in Ethiopia.

## Materials and methods

### Isolation, identification, and selection of strains

As part of a large epidemiological study, a total of 1,087 clinical samples (urine, stools, wound secretions, and sputum) were collected from patients with suspected infections seeking medical care from June to October 2016 at JMC, Ethiopia. *Pseudomonas* species and *Acinetobacter* species were isolated on MacConkey agar and sheep blood agar. Species identification was performed by MALDI-TOF (Bruker Daltonik GmbH, Bremen, Germany) at Karolinska University Hospital (KUH), Clinical Microbiology laboratory, and a full panel of antimicrobial susceptibility testing was performed by using the EUCAST 2021 v11 guideline.^1^

### Antimicrobial susceptibility testing

All *Pseudomonas* species and *Acinetobacter* species isolated were subjected to disk-diffusion susceptibility testing. Antibiotic discs of ceftazidime, meropenem, piperacillin-tazobactam, gentamicin, amikacin, ciprofloxacin was used for *Pseudomonas* species. Similarly, all *Acinetobacter* isolates were tested by using meropenem, imipenem, gentamicin, amikacin, ciprofloxacin, trimethoprim-sulfamethoxazole. Then, isolates with reduced susceptibility to ceftazidime and/or meropenem for *Pseudomonas* spp., and meropenem or imipenem for *Acinetobacter* spp. were selected for antimicrobial susceptibility testing using the newer antimicrobials (cefiderocol, ceftazidime-avibactam, ceftolozane-tazobactam, and imipenem-relebactam for *Pseudomonas* spp., and cefiderocol, meropenem, imipenem, and imipenem-relebactam for *Acinetobacter* spp. using microbroth dilution technique), and whole genome sequencing (WGS). Patients’ clinical data like admission, presence of underlying chronic illnesses, current use of antibiotics, and other factors was collected using a structured questionnaire.

### DNA extraction, whole genome sequencing and analysis of genomic data

Genomic DNA was extracted using Qiagen kits on EZ1 automated DNA extractions system. The extracted DNA was quantified using Qubit™ 3.0 (Massachusetts, United States) and library preps were performed using NEXTRA-kit (Illumina) and sequenced using HiSeq2500 (Illumina). Raw reads were assembled using SPAdes ver. 3.13.0, and the assembled draft genomes were used for querying different databases, MLST-typing 2.0, hosted at center for genomic epidemiology, and detection resistome profile by using ResFinder 4.1.^2^ ^,3^ Epidemiologic analysis of relatedness between the isolates, and to other international isolates was performed by the minimum spanning tree using the isolate genomes deposited at the public domain for *A. baumannii* at^4^, and *P. aeruginosa* at.^5^ The Genome sequences were deposited at the NCBI, SRA database (Bioproject number: PRJNA593604, Biosample accession: SUB11593554).

## Results

### Clinical and demographic characteristics of the patient

From a total of 1,087 non-repeat clinical samples collected during the study period, non-duplicate, non-fermenting Gram-negative bacilli that belong to either *Pseudomonas* spp. or *Acinetobacter* spp. were isolated from 80 patents. Most of these patients, 73.7% (59/80) were male, and 26.3% (21/80) were female. Ninety percent (72/80) of these patients were admitted to different wards in the hospital [surgical ward (*n* = 52), intensive care unit (*n* = 6), pediatric ward (*n* = 3), and medical ward (*n* = 11)]. Clinical samples collected include urine for urinary tract infections, sputum for lower respiratory tract infections, wound swab for wound infections/abscess, and stools for diarrhea. Most of these patients were admitted to the hospital for other underlying diseases and developed infection after admission, and most of them were treated with locally available antimicrobial therapy. Among *Pseudomonas* isolates 68.2% (28/41) were from surgical ward, followed by 17.0% (7/41) from medical ward, and 9.7% (4/41) from the intensive care unit (Table 1A). Similarly, 69.2% (27/39) of the *Acinetobacter* species were isolated from the surgical ward and 20.5% (8/39) from the medical ward (Table 1B).

**TABLE 1A:** Socio-demographic and clinical characteristics patients and isolation *Pseudomonas* species.

Patient ID	Age	Sex	Inpatient/ outpatient	Current diagnosis	*Current antibiotic	Specimen	Underlying disease	*Pseudomonas* species
I020	28	M	Inpatient	Surgical site infection	CRO, MET	Wound swab	Surgical incision	*P. aeruginosa*
I032	28	M	Inpatient	Urinary tract infection	CRO, MET	Urine	Trauma	*P. aeruginosa*
I038	30	M	Inpatient	Urinary tract infection	CRO	Urine	Severe head injury	*P. putida*
I043	17	M	Inpatient	Urinary tract infection	CRO	Urine	Aspiration pneumonia	*P. aeruginosa*
M019	70	M	Inpatient	COPD	CRO, VAN	Sputum	Cor pulmonale	*P. aeruginosa*
M030	60	M	Inpatient	Community-acquired pneumonia	CRO	Sputum	**COPD	*P. aeruginosa*
M074	50	M	Inpatient	COPD	VAN, CIP	Sputum	Asthma	*P. aeruginosa*
M119	40	M	Inpatient	Pneumonia	CRO	Sputum	Post TB fibrosis	*P. aeruginosa*
M304	40	M	Inpatient	Community-acquired pneumonia	No	Sputum	Post TB fibrosis	*P. aeruginosa*
M334	35	F	Inpatient	Severe community-acquired pneumonia	CRO	Sputum	No	*P. aeruginosa*
M521	60	F	Outpatient	Community-acquired pneumonia	CRO	Sputum	**T2DM	*P. aeruginosa*
P014	14	M	Inpatient	Wound infection	AUG	Wound swab	No	*P. aeruginosa*
P109	4	F	Inpatient	Diarrhea	AMOX, GENT	Stool	SAM, pneumonia	*P. aeruginosa*
S007	32	M	Inpatient	Necrotizing fasciitis	No	Wound swab	No	*P. aeruginosa*
S010	5	M	Inpatient	Wound infection	CAF, CLO	Wound swab	Trauma	*P. aeruginosa*
S011	7	M	Inpatient	Wound infection	AMP, CAF	Wound swab	Trauma	*P. aeruginosa*
S017	18	M	Inpatient	Necrotizing fasciitis	CRO, MET	Wound swab	No	*P. aeruginosa*
S019	60	M	Inpatient	Foot ulcer	CRO, MET	Wound swab	Diabetes mellitus	*P. aeruginosa*
S020	30	M	Inpatient	Wound infection	CRO, MET	Wound swab	Trauma	*P. aeruginosa*
S036	17	F	Inpatient	Wound infection	No	Wound swab	Unstable pelvis fracture	*P. aeruginosa*
S047	10	M	Inpatient	Wound infection	AMP	Wound swab	Chronic osteomyelitis	*P. aeruginosa*
S048	15	M	Inpatient	Wound infection	AMP, CAF	Wound swab	Fracture of femoral shaft	*P. aeruginosa*
S077	3	M	Inpatient	Wound infection	AMP, GENT	Wound swab	Colostomy, imperforation	*P. aeruginosa*
S116	50	F	Inpatient	Wound infection	AMOX, MET	Wound swab	Uterine cancer	*P. aeruginosa*
S129	77	M	Inpatient	Wound infection	CRO, VAN	Wound swab	Amputation of leg	*P. aeruginosa*
S133	45	M	Inpatient	Wound infection	CRO, MET	Wound swab	Neck injury trauma	*P. aeruginosa*
S114	25	F	Inpatient	Acute kidney infection	CRO, MET	Urine	Rib fracture	*P. aeruginosa*
S155	21	F	Inpatient	Wound infection	AMP, CAF	Wound swab	Infected skin graft	*P. aeruginosa*
S174	60	M	Inpatient	Wound infection	CAF, AMP	Wound swab	Infected fracture site	*P. aeruginosa*
S192	75	M	Inpatient	Urinary tract infection	CRO	Urine	**BOO	*P. putida*
S195	21	F	Inpatient	Wound infection	CRO, MET	Wound swab	Skin graft infection	*P. aeruginosa*
S198	57	M	Outpatient	Wound infection	CLO, CAF	Wound swab	Left femoral fracture	*P. aeruginosa*
S209	18	F	Inpatient	Wound infection	CRO	Wound swab	Burn wound	*P. aeruginosa*
S248	20	F	Inpatient	Contaminated wound		Wound swab	No	*P. aeruginosa*
S288	50	M	Inpatient	Pneumonia	No	Sputum	Abdominal mass	*P. aeruginosa*
S319	40	M	Inpatient	Urinary tract infection	No	Urine	BPH	*P. aeruginosa*
S325	37	M	Inpatient	Wound infection	CAF, AMP	Wound swab	Compound distal fracture	*P. aeruginosa*
S328	60	M	Inpatient	Pneumonia	CRO, MET	Sputum	3rd degree burn	*P. aeruginosa*
S332	30	M	Inpatient	Wound infection	CRO, MET	Wound swab	2nd degree burn	*P. fulva*
S356	30	M	Inpatient	Wound infection	CRO, MET	Wound swab	Infected palate of right knee	*P. aeruginosa*
S371	64	M	Inpatient	Surgical site infection	CLO, CAF	Wound swab	Laparotomy	*P. aeruginosa*

*Current antibiotics: CRO, ceftriaxone; Met, metronidazole; VAN, vancomycin; CLO, cloxacillin; CAF, chloramphenicol; CIP, ciprofloxacin; AMP, ampicillin; GENT, gentamicin.

**Underlying diseases: COPD, congestive obstructive pulmonary disease; T2DM, type-2 diabetes mellitus; BOO, Bladder outlet obstruction; BPH, Benign prostatic hyperplasia.

**TABLE 1B:** Socio-demographic and clinical characteristics patients and isolation *Acinetobacter* species.

Patient ID	Age	Sex	Inpatient/ outpatient	Current diagnosis	*Current antibiotic	Specimen	Underlying disease	Bacterial species
I027	45	F	Inpatient	Aspiration pneumonia	CRO, MET	Sputum	Stroke/hemiparalysis	*A. baylyi*
I030	28	F	Inpatient	Skin infection	No	Wound swab	No	*A. baumannii*
M029	22	M	Inpatient	Urinary tract infection	CRO	Urine	Retroviral infection	*A. baumannii*
M057	60	F	Inpatient	COPD	No	Sputum	No	*A. baumannii*
M135	70	F	Outpatient	Community-acquired pneumonia	CRO	Sputum	No	*A. junii*
M212	25	M	Inpatient	Pneumonia	CRO, VAN	Sputum	Disseminated tuberculosis	*A. calcoaceticus*
M217	35	M	Inpatient	Community-acquired pneumonia	No	Sputum	**COPD	*A. schindleri*
M328	50	F	Outpatient	Severe community-acquired pneumonia	No	Sputum	**T2DM	*A. baumannii*
M344	17	M	Outpatient	Pneumonia	No	Sputum	Electrical burn	*A. junii*
M431	50	F	Inpatient	Pneumonia	CRO	Sputum	Rt&Lt femoral fracture	*A. baumannii*
P015	30	M	Inpatient	Wound infection	CLO, CAF	Wound swab	Compound fracture of right tibia	*A. baumannii*
P038	35	M	Outpatient	Wound infection	AMP, CAF	Wound swab	Infected fracture site	*A. baumannii*
S073	15	M	Inpatient	Wound infection	CRO	Wound swab	Chronic osteomyelitis	*A. baumannii*
S082	45	M	Inpatient	Wound infection	CIP	Wound swab	Femoral fracture	*A. baumannii*
S126	40	M	Inpatient	Wound infection	CRO, MET	Wound swab	Surgical site infection	*A. baumannii*
S130	30	M	Inpatient	Wound infection	CRO, MET	Wound swab	3rd deg. burn	*A. baumannii*
S147	30	F	Inpatient	Wound infection	CRO	Wound swab	Colostomy	*A. baumannii*
S161	50	M	Inpatient	Wound infection	AMP, CAF	Wound swab	No	*A. parvus*
S165	8	M	Inpatient	Wound infection	CAF, CLO	Wound swab	No	*A. baumannii*
S167	40	M	Inpatient	Wound infection	CRO, MET	Wound swab	Scalp abscess	*A. baumannii*
S170	27	M	Inpatient	Wound infection	CRO, MET	Wound swab	No	*A. baumannii*
S171	32	M	Inpatient	Wound infection	CRO, MET	Wound swab	Retroviral infection	*A. baumannii*
S176	25	F	Inpatient	Necrotic wound infection	CRO, MET	Wound swab	No	*A. baumannii*
S209	50	M	Outpatient	Pneumonia	CRO, MET	Sputum	No	*A. baumannii*
S210	25	M	Inpatient	Wound infection	CRO	Wound swab	No	*A. baumannii*
S212	40	M	Inpatient	Wound infection	CRO, MET	Wound swab	Compound fracture left leg	*A. baumannii*
S217	48	M	Inpatient	Pneumonia	CRO, MET	Sputum	colostomy/post-operation	*A. baumannii*
S219	39	M	Inpatient	Wound infection	No	Wound swab	Tibia-fibular fracture	*A. baumannii*
S226	24	M	Inpatient	Urinary tract infection	No	Urine	Urethral stricture	*A. haemolyticus*
S247	40	M	Outpatient	Wound infection	AMP, CAF	Wound swab	Infected incision site	*A. baumannii*
S260	18	F	Inpatient	Wound infection	CRO	Wound swab	Burn wound	*A. baumannii*
S267	30	F	Inpatient	Wound infection	CRO, MET	Wound swab	Surgical site infection	*A. baumannii*
S270	26	M	Inpatient	Wound infection	CRO, MET	Wound swab	Chronic osteomyelitis	*A. baumannii*
S275	48	M	Inpatient	Wound infection	No	Wound swab	Compound fracture of femur	*A. baumannii*
S294	70	M	Inpatient	Urinary tract infection	CRO	Urine	Bladder outlet obstruction	*A. baumannii*
S296	10	M	Inpatient	Wound infection	AMP, CAF	Wound swab	Left femoral fracture	*A. baumannii*
S315	2	M	Inpatient	Diarrhea	AMP, GENT, CLO	Stool	No	*A. lwoffii*
S327	29	M	Inpatient	Severe community-acquired pneumonia	CRO, MET	Sputum	No	*A. baumannii*
S347	18	F	Inpatient	Urinary tract infection	AMP, CAF	Urine	Cervical Spine fracture	*A. baumannii*

*Current antibiotics: CRO, ceftriaxone; Met, metronidazole; VAN, vancomycin; CLO, cloxacillin; CAF, chloramphenicol; CIP, ciprofloxacin; AMP, ampicillin; GENT, gentamicin.

**Underlying diseases: COPD, congestive obstructive pulmonary disease; T2DM, type-2 diabetes mellitus.

### Species diversity and antimicrobial susceptibility

#### Species diversity

From a total of 41 isolates of *Pseudomonas* spp., 92.6% (38/41) were *P. aeruginosa*, and 7.3% (3/41) were another *Pseudomonas* spp. Among 39 isolates of *Acinetobacter* spp. (*n* = 39), 79.5% (31/39) were *A. baumannii*, and 20.5% (8/39) were other *Acinetobacter* spp. [*A. junii* which account for 5.1% (2/39), and *A. baylyi, A. lwoffii, A. calcoaceticus, A. haemolyticus, A. parvus*, and *A. schindleri* each account for 2.5% (1/39)].

#### Antimicrobial susceptibility pattern

Among isolates of *Pseudomonas* spp., 31.7%, (13/41) were non-susceptible to ceftazidime, and 7.3% (3/41) to meropenem. Among isolates of *Acinetobacter* spp., 56.4% (22/39) were carbapenem-resistant. Most of the ceftazidime-resistant *Pseudomonas* spp. were also resistant to piperacillin-tazobactam 84.6% (11/13), ciprofloxacin 30.7% (4/13), ceftazidime-avibactam 46.1% (6/13) and ceftolozane-tazobactam 53.8% (7/13). However, most of these isolates were susceptible to cefiderocol 84.7% (11/13), imipenem-relebactam 92.3% (12/13), and all *Pseudomonas* isolates were susceptible to amikacin (Table 2A). Most of the *Acinetobacter* isolates were resistant to meropenem, imipenem, and imipenem-relebactam 54.5%, (12/22). However, a lower rate of resistance was detected to ciprofloxacin 18.1% (4/22), gentamicin 27.2% (6/22), amikacin 18.1% (4/22), and cefiderocol 9.1% (2/22) (Table 2B).

**TABLE 2A:** Antimicrobial susceptibility pattern of ceftazidime resistant *Pseudomonas* spp. isolated at JMC, Ethiopia.

Bacterial species		Type of antimicrobials used for antimicrobial susceptibility testing									
		Ceftazidime	Meropenem	Imipenem	Piperacillin-tazobactam	Ciprofloxacin	Amikacin	Cefiderocol	Ceftazidime-avibactam	Ceftolozane-tazobactam	Imipenem-relebactam
*Pseudomonas aeruginosa* (*n* = 10)	S (%)	0 (0.0%)	7 (70)	8 (80)	0 (0)	7 (70)	10 (100)	9 (90)	5 (50)	6 (60)	9 (90)
	R (%)	10 (100)	3 (30)	2 (20)	10 (100)	3 (30)	0 (0)	1 (10)	5 (50)	4 (40)	1 (10)
Other *Pseudomonas* species *(n* = *3)*	S (%)	1 (32.4)	1 (32.4)	3 (100)	1 (32.4)	2 (66.6)	3 (100)	2 (66.6)	2 (66.6)	0 (0)	3 (100)
	R (%)	2 (66.6)	2 (66.6)	0 (0)	2 (66.6)	1 (32.4)	0 (0)	1 (32.4)	1 (32.4)	3 (100)	0 (0)

S, susceptible; R, resistant, other *Pseudomonas* species [*Pseudomonas putida* (*n* = 2), *Pseudomonas fulva* (*n* = 1)].

**TABLE 2B:** Antimicrobial susceptibility pattern of carbapenem resistant *Acinetobacter* species isolated at JMC, Ethiopia.

Bacterial species		Type of antimicrobials used for antimicrobial susceptibility testing							
		Meropenem	Imipenem	Imipenem-relebactam	Cefiderocol	Ciprofloxacin	Trimethoprim/sulfamethoxazole	Gentamicin	Amikacin
*Acinetobacter baumannii* (*n* = 22)	S (%)	5 (27.8)	9 (50)	9 (50)	17 (94.4)	14 (77.8)	12 (66.7)	12 (66.7)	15 (83.3)
	R (%)	13 (72.2)	9 (50)	9 (50)	1 (5.6)	4 (22.2)	6 (33.3)	6 (33.3)	3 (16.7)
Other *Acinetobacter* spp.	S (%)	1 (25)	1 (25)	1 (25)	4 (100)	1 (25)	0 (0)	4 (100)	4 (100)
	R (%)	3 (75)	3 (75)	3 (75)	0 (0)	3 (75)	4 (100)	0 (0)	(100)

S, susceptible; R, resistant; Other *Acinetobacter* spp. (*n* = 4). (*A. calcoaceticus, A. baylyi, A. junii*, and *A. lwoffii*).

### Resistome profile of *Pseudomonas* and *Acinetobacter* species

Resistome profiling showed that both *Pseudomonas* and *Acinetobacter* spp. encoded multiple β - lactamase genes. The *bla*_OXA–486_, *bla*_OXA–50_ and *bla*_CTX–M–15_ genes were most common among *Pseudomonas* isolates, and the *bla*_OXA–51_-like *bla*_OXA–69,_ *bla*_OXA–66,_ *bla*_OXA–91,_ *bla*_OXA–180_ and *bla*_GES–11_ were the most common among *Acinetobacter* isolates (Table 3A).

**TABLE 3A:** Sequence types and resistance genes observed among carbapenem-resistant Acinetobacter species isolated at Jimma Medical Center, Ethiopia (*n* = 19).

Isolate ID	STs	Number and type of antimicrobial resistance genes						
		Carbapenemase	ESBL and other β -lactamases	Aminoglycosides	Trimethoprim	Sulfonamides	Tetracycline	Macrolides
I027AB*	ST2	*bla*_OXA–69_	*bla*_GES–11_	*aac(6′)-Ib3, aac(6′)-Ib-cr*	*dfrA7*	*sul1*		
I030AB	ST1	*bla*_OXA–69_	*bla*_GES–11_	*aac(6′)-Ib3, aac(6′)-Ib-cr*	*dfrA7*	*sul1*		
M217AB	ST1	*bla*_OXA–69_	*bla*_GES–11_	*aac(6′)-Ib3, aac(6′)-Ib-cr*	*dfrA1*,	*sul1*		
M328AB	164	*bla*_OXA–91_	*bla*_CARB–5_, *bla*_CARB–49_				*tet(39)*	
P015AL	NA		*bla*_CTX–M–15,_ *bla*_OXA–1_,	*aac(6′)-Ib-cr*			*tet(A)*	*mdf(A), mph(A), mph(D)*
S126AB	ST2090	*bla*_OXA–259/180_						
S130AB	ST85	*bla*_NDM–1_, *bla*_OXA–94_		*ant(2″)-Ia, aph(3′)-VI*		*sul2*		*mph(E) msr(E)*
S161AB	ST2	*bla*_OXA–66_	*bla*_TEM–1D_,	*aac(3)-Ia, aph(6)-Id*		*sul1*		
S167AB	ST1	*bla*_OXA–69_	*bla*_GES–11_	*aac(6′)-Ib-cr, aac(6′)-Ib3*	*dfrA7*	*sul1*		
S170AB	ST164	*bla*_OXA–91_	*bla*_CARB–49_, *bla*_CARB–16_				*tet(39)*	
S171AB	ST1	*bla*_OXA–69_	*bla*_GES–11_	*aac(6′)-Ib3, aac(6′)-Ib-cr*	*dfrA7*	*sul1*		
S176AB	ST1	*bla*_OXA–69_	*bla*_GES–11_	*aac(6′)-Ib3, aac(6′)-Ib-cr*	*dfrA7*	*sul1*		
S209AB	ST2	*bla*_OXA–66_		*aac(3)-Ia, aadA1, aph(6)-Id*		*sul1sul2*	*tet(B)*	
S212AB	ST1	*bla*_OXA–69_	*bla*_GES–11_	*aac(6′)-Ib3, aac(6′)-Ib-cr*,	*dfrA7*	*sul1*		
S270AB	ST1	*bla*_OXA–69_	*bla*_GES–11_	*aac(6′)-Ib3, aac(6′)-Ib-cr*	*dfrA7*	*sul1*		
S275AB	ST1	*bla*_OXA–69_	*bla*_GES–11_	aac(6′)-Ib3, aac(6′)-Ib-cr	dfrA7	sul1		
S296AB	ST1	*bla*_OXA–69_	*bla*_GES–11_	aac(6′)-Ib3, aac(6′)-Ib-cr	dfrA7	sul1		
S315AB	ST1	*bla*_OXA–69_	*bla*_GES–11_	aac(6′)-Ib3, aac(6′)-Ib-cr	dfrA7	sul1		
S327AB	ST1	bla_OXA–69_						

Moreover, two isolates, *P. aeruginosa* (*n* = 1) and *A. baumannii* (*n* = 1) carrying the *bla*_NDM–1_ carbapenemase gene were detected. Furthermore, the resistome profile shows that resistance genes to the antimicrobial classes of aminoglycosides, fluoroquinolones, and trimethoprim were prevalent among these two isolates (Tables 3A,B).

**TABLE 3B:** Sequence types and resistance genes observed among ceftazidime-resistant *Pseudomonas* spp. isolated at Jimma Medical Center, Ethiopia (*n* = 13).

Strain ID	STs	Number and type of antimicrobial resistance genes						
		Carbapenemase	β -lactamase genes	Aminoglycosides	Fluoroquinolones	Sulfonamides	Phenicols	Fosfomycin
I020PA*	11		*bla*_OXA–486_, *bla*_PAO_	*aph (3′)-IIb*			*catB7*	*fosA*
I032PA	2948	*bla*_NDM–1_	*bla*_OXA–10_, *bla*_OXA–50_	*aadA1, ant(2″)-Ia, aph(3′)-IIb*	*crpP*	*sul1*	*catB7, cmlA1*	*fosA*
I038PP*	NA		*bla*_CTX–M–15_, *bla*_OXA–1_	*aac(6′)-Ib-cr*,				
M019PA	1228		*bla*_vEB–1_, *_*bla*_*_OXA–486_ *bla*_OXA–10_	*aadA1, aadA2 ant(2″)-Ia, aph(3′)-IIb*	*crpP qnrD1*	*sul1*	*catB7 cmlA1*	*fosA*
M119PA	274		*bla*_OXA–486_, *bla*_PAO_,				*catB7*	*fosA*
M304PA	274		*bla*_OXA–486_, *bla*_PAO_				*catB7*	*fosA*
S116PA	500		*bla*_OXA–486_, *bla*_PAO_		*crpP*		*catB7*	*fosA*
S114PP*	NA		*bla*_CTX–M–15_, *bla*_OXA–10_ *bla*_OXA–1_	*aac(3)-Iia, aac(3)-Ib aac(6′)-Ib, aac(6′)-Ib-cr, ant(3″)-Ia, aph(3″)-Ib*	*oqxB qnrVC1*	*sul1*		
S155PA	274		*bla*_OXA–486_, *bla*_PAO_	*aph(3′)-IIb*			*catB7*	*fosA*
S248PA	840		*bla*_OXA–486_, *bla*_PAO_	*aph(3′)-IIb*	*crpP*		*catB7*	*fosA*
S332PF**	NA		*bla*_PER–1_,	*aph(6)-Id*	*qnrVC6*	*sul1*		
S356PA	646		*bla*_CTX–M–15_, *bla*_OXA–494_ *bla*_OXA–50_, *bla*_TEM–1B_	*ant(2″)-Ia, aph(3′)-IIb aph(3′)-Ia, ph(6)-Id*		*sul1*	*catB7, floR*	*fosA*
S047PA	244		*bla*_OXA–50_	*aph(3′)-IIb*			*catB7*	*fosA*

*A. baumannii; PA, *Pseudomonas aeruginosa*; PP**, *Pseudomonas putida*; PF**, Pseudomonas fulva.

### Molecular epidemiology using cgMLST

Epidemiologic typing using the seven gene multi-locus sequence typing demonstrated that ST274 (23.0%, 3/13) among *P. aeruginosa* and ST1 (MLST-Pasteur) *A. baumannii* (63.1%, 12/19) were the most prevalent sequence types (Tables 3A,B). A cgMLST analysis showed that the ST1 *A. baumannii* isolates were highly similar with no allelic differences between them. Most of the *Acinetobacter* isolates belonged to the international clonal complexes, ICC1 (includes ST1) (*n* = 12) and ICC2 (*n* = 2) (Table 3A).

## Discussion

In this study, nearly all isolates of both *Pseudomonas* and *Acinetobacter* spp. were from patients admitted to the hospital for more than 72 h. Nosocomial acquisition of MDR isolates of *Pseudomonas* spp. and *Acinetobacter* spp. is worrisome. The situation is complicated by limited availability of antimicrobial agents, lack of prescription guidelines, and insufficient standard routine microbiology laboratory services to support antibiotic selection. In such cases, the safety of patients admitted to the hospital can be severely compromised.

The prevalence of ESBL-producing strains among *P. aeruginosa*, and carbapenem non-susceptible isolates among *A. baumannii* strains was high in the present study. Previous phenotypic studies from Ethiopia also show that MDR strains of *Pseudomonas* and *Acinetobacter* were prevalent (Motbainor et al., 2020; Birhane Fiseha et al., 2021). However, most of these studies did not describe the genotypes and resistome profile of the isolates, and mechanism of resistance is difficult to compare between studies. So far, to our knowledge, there is no report of a genotypic study on the prevalence of ESBL-producing *P. aeruginosa* in Ethiopia. Generally, there are limited studies conducted on carbapenemase-producing *P. aeruginosa* in Africa. The few reports available are from northern Africa and mainly from Egypt (Osei Sekyere and Reta, 2020; Rizk et al., 2021). In northern Africa, the prevalence ranges from 0 to 96% (Gaballah et al., 2018). A finding from Uganda (7.4%) was comparable to the present study (Aruhomukama et al., 2019). But, one study from South Africa (51.0%) reported a higher prevalence of ceftazidime resistant *P. aeruginosa* compared to the present study (Hosu et al., 2021). The phenotypic studies from Ethiopia, and other African countries showed higher prevalence as compared to the present study.

In Ethiopia, a high prevalence of carbapenem-resistant *Acinetobacter* spp. was reported from one previous phenotypic study (Ayenew et al., 2021), which is comparable to the prevalence of carbapenem resistance herein. On the other hand, a systematic review and meta analysis on carbapenemase producing *P. aeruginosa* and *A. baumannii* in Africa showed that the lowest prevalence of carbapenemase-producing *A. baumannii* was 4.7% (*n* = 21), and the highest prevalence was 100% (*n* = 7) (Kindu et al., 2020). Studies conducted on *P. aeruginosa* and *A. baumannii* strains from Africa were limited to small sample sizes and were mainly phenotypic studies, which makes the comparison with genotypic studies difficult (Kindu et al., 2020; Osei Sekyere and Reta, 2020; Ayenew et al., 2021; Mekonnen et al., 2021). However, most available studies including a study for hospital environment (Solomon et al., 2017) reported higher prevalence of the MDR *P. aeruginosa* and *A. baumannii* in Ethiopia, calling for the application of genotypic methods to studies on mechanisms of resistance and spread.

The multiple genetic variants of antibiotic resistance observed among both *Pseudomonas* and *Acinetobacter* spp. pose a huge challenge on the limited therapeutic options available to low-income countries. Among *Acinetobacter* spp., the presence of the *bla*_GES–11_ (ESBL-genotype and weak carbapenemase) and the OXA-51-like (*bla*_OXA–66_ and *bla*_OXA–69_) carbapenemases encoding genes is a serious threat. The OXA-51-like intrinsic carbapenemase encoding ST1 *A. baumannii* Isolates were reported from India (Rose et al., 2021), but isolates from the present study encoded additionally the weakly carbapenem hydrolyzing *bla*_GES–11_ gene. The *bla*_GES–11_ ESBL-genotypes, and the *bla*_OXA–51_-like carbapenemases have not been previously reported from Ethiopia, and the present study is to our knowledge the first report of *bla*_OXA–51_ like and *bla*_GES–11_ from Ethiopia. Generally from Africa, only one study from Tunisia has documented clinical isolates *of A. baumannii* encoding the *bla*_GES–11_ (Chihi et al., 2016). The emergence of *bla*_NDM–1_ encoding isolates of *A. baumannii* at surgical ward, and *P. aeruginosa* at intensive care unit can severely compromise the safety of vulnerable patients admitted to the hospital. Moreover, detection of two isolates encoding the *bla*_NDM–1_ which showed resistance to the newer antimicrobials, *A. baumannii* for (cefiderocol and imipenem-relebactam), and *P. aeruginosa* for (cefiderocol, ceftazidime-avibactam, ceftolozane-tazobactam) compromises the already limited treatment alternatives for vulnerable groups of patients at this hospital.

**FIGURE 1:**
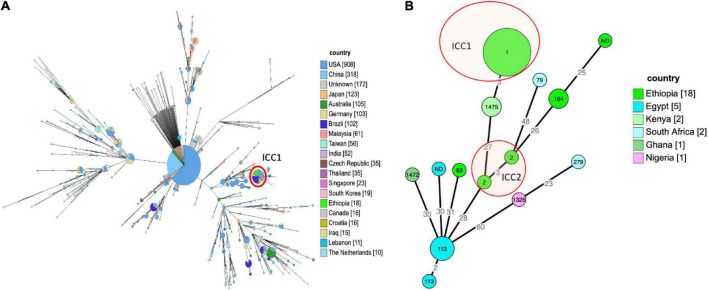
**(A)** Epidemiologic relatedness of *A. baumannii* isolates to the isolates collected from different geographical regions and deposited in the public database (pubmlst https://pubmlst.org/organisms/acinetobacter-baumannii~-- accessed on 26-09-2021), the isolates from this study (bright green) were clustered with the global ICC1 clone marked with red circle. The numbers in the circle show the sequence type of the isolates, the size of the circle is proportional to the number of isolates that belongs to that sequence type, and the color shows the country of origin for the isolates. The minimum spanning tree was constructed for isolates from different country of origin having ≥ 10 isolates recorded in the database. **(B)** Minimum spanning tree of 29 *A. baumannii* isolates from Africa, the tree was constructed based on core genome multi-locus sequence typing. There were no previous isolates from Ethiopia in PubMLST, all isolates labeled Ethiopia in the tree are from this study.

Sequence typing showed that both *P. aeruginosa* and *A. baumannii* strains were polyclonal. But, since a large proportion of *A. baumannii* isolates were ST1, the spread of *A. baumannii* isolates at this hospital might also be to some extent clonal. A previous study conducted at the same hospital had identified three strains of *A. baumannii* encoding *bla*_NDM–1_, and all of them belonged to the ST597. Furthermore, cgMLST analysis of *A. baumannii* with other international isolates in pubmlst showed that isolates in the current study were distinct from isolates from other African countries (Figure 1).^6^ However, these isolates were clustered with isolates from other countries like United States, and Brazil. Though isolates from this study were polyclonal, the most prevalent isolates were those that belong to the international cluster, CC1 and CC2.

**FIGURE 2:**
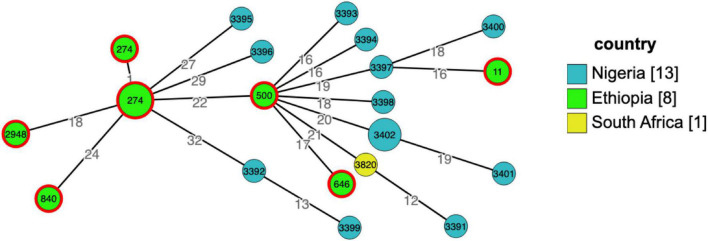
Grape tree generated using cgMLST from genomic collection of *Pseudomonas aeruginosa* isolates from Africa (*n* = 22), Ethiopia, Nigeria, and South Africa, pubmlst database for microorganisms (https://pubmlst.org/bigsdb?db=pubmlst_paeruginosa_isolates&page=query&genomes=1) accessed on 29-11-2021. The numbers in circles/nodes indicate sequence types of the isolates, size of the nodes is proportional to the number of isolates in a particular sequence type and the numbers on the branches are the number of allelic differences between the two neighboring nodes. From 10 isolates sequenced in this study, eight were included in the tree, two of the sequences failed quality required to upload to the pubmlst database.

Although *A. baumannii* is a well-known major cause of nosocomial infections, knowledge of its genomic epidemiology and availability of reliable data regarding the genetic basis of antibiotic resistance is limited in low-income countries. Similarly, *P. aeruginosa* isolates were found to be polyclonal, and different from a collection of isolates other African counties found in pubmlst (see text footnote 5) (Figure 2).

The current study may serve as a baseline regarding local spread of international clones and alert clinicians and other health workers, researchers, and public health policy makers to the problem. Implementation of strict infection prevention and control strategies, and antimicrobial stewardship programs are highly desirable in the admission wards where the international clones are spreading. Furthermore, despite limitation of resources, the added value of next generation sequencing is in understanding the dynamics and mechanisms of spread of MDR bacterial clones.

## Conclusion

The prevalence of MDR isolates is high among both in clinical isolates of *Pseudomonas* species and *Acinetobacter* species at Jimma medical center. Emergence of the *bla*_NDM–1_ in clinical isolates of *P. aeruginosa* and *A. baumannii* strains is worrisome. However, the susceptibility of *P. aeruginosa* strains to amikacin, cefiderocol, imipenem-relebactam and ceftolozane-tazobactam, and *A. baumannii* strains to amikacin and cefiderocol is important to consider as alternative options to carbapenems. The use of next generation sequencing is important to understand the mechanism of resistance and spread of resistant clones such as ICC1, and ICC2 *A. baumannii* strains detected at this hospital.

## Data availability statement

The genome sequences were deposited at the NCBI, SRA database (PRJNA593604, Biosample accession: SUB11593554).

## Ethics statement

The study obtained ethical approval from Addis Ababa University Institutional Review Board (AAU-IRB), Armauer Hansen Research Institute – ALERT Hospital Institutional Review Board (AHRI-ALERT-IRB), and Ethiopian National Ethics Review Committee (NERC). Patients were informed about the study and given written consent to participate in the study.

## Author contributions

TS contributed to design, data acquisition, data analysis, and drafting of the manuscript. DA and YW contributed to data acquisition and write-up. AA contributed to the design, data acquisition, and revision of the manuscript. CG contributed to the overall design, data acquisition, supervision, drafting, and writing of the manuscript. All authors contributed to the article and approved the submitted version.

## Footnotes

1
www.eucast.org/fileadmin/src/media/PDFs/EUCAST_files/Breakpoint _tables/v_11.0_Breakpoint_Tables.pdf

2
https://cge.food.dtu.dk/services/MLST/

3
https://cge.food.dtu.dk/services/ResFinder/

4
https://pubmlst.org/bigsdb?db=pubmlst_abaumannii_isolates&page=query&genomes=1

5
https://pubmlst.org/organisms/pseudomonas-aeruginosa

6
https://pubmlst.org/organisms/acinetobacter-baumannii
